# Suicide and sudden violent death among young people: Two sides of the same coin?

**DOI:** 10.1371/journal.pone.0313673

**Published:** 2024-12-04

**Authors:** Annelie Werbart Törnblom, Andrzej Werbart, Kimmo Sorjonen, Bo Runeson

**Affiliations:** 1 Department of Women’s and Children’s Health, Karolinska Institutet (KI), Stockholm, Sweden; 2 Department of Psychology, Stockholm University, Stockholm, Sweden; 3 Division of Psychology, Department of Clinical Neuroscience, Karolinska Institutet (KI), Stockholm, Sweden; 4 Department of Clinical Neuroscience, Centre for Psychiatry Research, Karolinska Institutet (KI), Stockholm County Council, Stockholm, Sweden; Università degli Studi di Torino: Universita degli Studi di Torino, ITALY

## Abstract

The aim of the present study was to compare risk factors for death by suicide and sudden violent death (SVD) among young people aged 10–25 years. Two target samples, 63 consecutive cases of youth suicide and 62 cases of SVD, were compared on potential risk factors differentiating the two groups from 104 controls. Data on psychiatric diagnoses, psychosocial factors, adverse childhood experiences, stressful life events, and coping strategies were collected in psychological autopsy interviews. Distinguishing for the suicide group was lower frequency of living in a steady relationship, adult psychiatric care, depression, autism spectrum disorder, being sexually assaulted, higher frequency of recent stressful life events, and lowest levels of adaptive coping. Distinguishing for the SVD group was a predominance of males, lower elementary school results, abuse of psychoactive drugs, being investigated or sentenced for criminal acts, conduct disorder or antisocial personality disorder. Common risk factors for both kinds of premature unnatural death included lower educational level, absence of work or studies, different forms of addiction, child and adolescent psychiatric care, borderline personality disorder, adverse childhood experiences, and less adaptive coping. Accordingly, there is a common ground of vulnerabilities, early adversities, and recent strains in life for both forms of premature death, but also substantial differences between these contrasting lethal developments. Prevention of both suicide and SVD should focus on adverse childhood experiences, learning difficulties, meaningful occupation, more adaptive coping, addiction, and treatment of borderline personality disorder. Suicide prevention should comprise promotion of adaptive stress management skills, depression prevention and treatment, and paying attention to young people with autism. SVD prevention should involve early response to learning difficulties, abuse of psychoactive drugs and delinquent behavior, and treatment of conduct disorder and antisocial personality disorder.

## Introduction

Suicide and different forms of sudden violent death (SVD) are the most common causes of death among young people worldwide. The phrase “young people,” the target group for our investigation, is generally used in Sweden and the Anglo-Saxon countries as a generic term for children, adolescents, and young adults. To better understand young people who kill themselves or expose themselves to risks resulting in SVD, we need to know more about the interplay between adverse life events, both in the far past and present, vulnerability factors, and coping deficits that may result in such lethal outcomes.

Suicide is defined by the World Health Organization [1] as “the act of deliberately killing oneself,” and a suicide attempt as “any non-fatal suicidal behaviour and refers to intentional self-inflicted poisoning, injury or self-harm which may or may not have a fatal intent or outcome.” The report warns that it can be difficult to distinguish between self-harm with or without suicidal intent, suggesting complexities in demarcating suicide and SVD.

In psychological terms, the process behind overt suicide and other forms of self-destructive behavior is often interpreted as hostile impulses winning over self-protecting forces. For example, Menninger [2] regarded self-destructive acts as inwards-directed aggressiveness. Furthermore, he described abortive, distorted, or attenuated forms of “latent suicide” as result of indirect or incomplete self-destructive behavior, such as addiction or accidents. According to Goldbladt’s [3] more recent psychoanalytic perspective, suicide takes place in the interpersonal and intrapsychic context of unbearable hostility towards the self. Accordingly, a French prospective epidemiological cohort study [4] found that increased risk of suicide is related to cognitive rather than behavioral hostility. The currently often cited Columbia—Suicide Severity Rating Scale (C-SSRS) [5] includes a definition of actual attempt, interrupted attempt, aborted or self-interrupted attempt or preparatory act or behavior to define different levels of suicidal behavior. Accordingly, several authors [6, 7] described a continuum of self-destructive behavior among younger age groups from covert expressions to overt suicidal behavior.

According to current reviews, common risk factors for suicide from childhood to young adulthood include genetic and epigenetic factors, early life adversities, lack of social support, life events, access to lethal means, effects of the media, severe mental illness, depression, personality disorder, substance misuse, economic factors, and physical health problems [8], as well as history of suicidal behavior and psychiatric care [9–11]. A systematic review [12] found evidence for an association between life stressors, particularly interpersonal stressors, and death by suicide. As summarized in an overview of psychological models of suicide [13], some models focus on vulnerability factors, such as “impulsive aggressive tendencies, maladaptive cognitive styles, problem solving deficits, attention bias, over-general memory, and acquired capability for self-harm,”, whereas other models emphasize the role of stressful life events, leading to “mental pain, hopelessness, entrapment, and interpersonal distress” (p. 306). However, few theoretical models and empirical studies try to integrate these two clusters of factors or grasp the interactions between different factors.

Major risk factors for SVD among young people, identified in several studies [14–17], include being of male sex, antisocial personality disorder, criminality, alcohol and drug abuse, adverse family psychosocial characteristics, as well as aggressive feelings and acts against oneself and others, health-compromising behavior, and risk-taking behavior. Thus, there are some similarities but also several differences between risk factors for suicide and SVD among youth. Accordingly, the two-stage model of suicide and violence [13] assumes that both suicide and SVD are expressions of the same underlying aggressive impulse. Hence, other intervening variables determine whether the aggression is directed inward or expressed in outward-directed behavior.

Major protective factors for youth suicidality, found in empirical studies [18, 19] and reviews [20, 21], include parental presence, connectedness to parents and peers, belongingness to community and social institutions, positive connection to school and academic achievement, social competence, coping and problem-solving skills, contacts with caregivers, and effective mental health care. Much less is known about protective factors against SVD. A systematic review [22] found that adaptive coping has a protective function against stress and is related to well-being in the transition to adulthood. Thus, adequate coping skills may be expected to have protective function against both suicide and SVD.

In a previous study [23], we examined associations between stressful life events and coping strategies in cases of youth suicide and in cases of youth SVD, as compared to general population control cases. We found that between-group differences in coping were partly accounted for by differences in negative life events, early and later in life. In the present study we explore the assumption that there may be both common factors, distinguishing children, adolescents, and young adults who died by suicide or SVD from the general population, and specific factors that are unique for the two groups, comparing the two target groups on variables significantly distinguishing them from control cases. A review [24] found that the term “sudden violent death” usually includes death by suicide, accident, homicide, or overdose. In the present study, suicide is defined as the act of deliberately killing oneself, and SVD as unintentional injury-related (that is, non-suicidal) death, which may still have occurred due to an underlying, hidden intention to die. As far as we know, there is a scarcity of studies and lack of reviews exploring common paths and dividing developments for suicide and SVD among children and young people.

## Methods

### Study design and sample

The present study is based on archival interview data collected from May 28, 2001, to January 28, 2008, in an investigation of suicide and SVD (murder, accident, unclear accident) up to the age of 25 years in Stockholm County, Sweden, and including a control group. Consequently, the sample and some of the data presented here have already been used in previous publications [23, 25]. The project was approved by the Regional Ethical Review Board, Karolinska Institutet, Stockholm (reference number 96–204 and 2005/530-32), and for the control group also the Swedish State Personal Address Register (reference number 2004/0146). All informants (parents and relatives of the deceased, as well as all participants in the control group and their parents) gave written informed consent.

In order to compute the required sample size a priori power analysis was conducted applying G*Power [26]. For a one-way ANOVA with three groups, 0.05 alpha level, effect size f = 0.25 (medium effect size corresponding to Cohen’s *d* = 0.5), and power 0.8, the required sample size per group is 53. With 60 persons per group, the achieved power will be 0.85.

Consecutive cases of non-natural death among children, adolescents, and young adults were identified at the Department of Forensic Medicine in Stockholm, which is responsible for all forensic autopsies in the Stockholm Region. Information on causes of death was based on autopsy protocols and police reports. Consecutive cases of suicide were collected from October 6, 2000, through December 30, 2004, and the consecutive cases of SVD from October 1, 2000, through September 11, 2002. Such a long time was required to achieve the target number of at least 60 cases in both groups. The control cases were collected from a randomized sample obtained from the population registry in Stockholm County (data collected from January 18, 2006, through January 28, 2008). Looking for risk factors for suicide and SVD, the control group was originally matched with the two target groups taken together on two variables: gender and age, according to a review [27] the most common procedure in psychological autopsy studies. No other matching criteria were used, as we wanted to include a wide range of potential risk factors, such as strains in life and socio-demographic characteristics.

In the present study, the anonymized database was accessed from July 6, 2019. All interview data were collected by the first author who unavoidably had access to information that could identify individual participants during the data collection. None of the other authors had access to such information during or after data collection.

Two target samples and a control group were included (for sociodemographic characteristics and study variables, see Table 1). Of the 63 cases of suicide (aged 12–25 years), 41 were males and 22 females. Seven of the cases of suicide (11%) were younger than 18 years. Of the 62 cases of SVD (aged 10–25 years), 55 were males and 7 females. Ten of the cases of SVD (16%) were younger than 18 years. Both target groups, taken together, included 125 cases of premature unnatural death; 96 males (77%) aged 10–25 years (*M* = 21.0; *SD* = 3.2*; Md* = 22) and 29 females (23%) aged 14–24 years (*M* = 19.8; *SD* = 3.0; *Md* = 22). This can be compared with the 104 control cases, of which 76 were males (73%) aged 10–25 years (*M* = 20.7; *SD* = 3.4; *Md* = 21) and 28 were females (27%) aged 14–24 years (*M* = 19.7; *SD* = 3.0; *Md* = 20). In all, 229 families took part in the study (among the control families, the individual young person was included in the interview).

**Table 1:** Sociodemographic, psychosocial and psychiatric data, and univariate effects of the potential risk factors on the dependent variable.

Variable	Suicide		Sudden violent death		Suicide vs. sudden violent death			
	*N* = 63	%	*N* = 62	%	OR	95% CI	p-value	
Gender (female)	22	34.9	7	11.3	4.216	1.644	10.812	0.003
Males age	M = 21.4 (12–25)	SD = 2.5	M = 20.7 (10–25)	SD = 3.7				
Females age	M = 19.7 (14–24)	SD = 3.3	M = 20.0 (17–22)	SD = 1.9				
Mother’s age at the child’s birth	M = 29.7 (16–42)	SD = 5.8	M = 27.1* (17–39)	SD = 5.5	1.084	1.016	1.157	0.015
Elementary school,^1^ average or higher grades	51^4^	83.6	43***	69.4	3.813	1.603	9.071	0.002
Upper secondary school, average or higher grades	31*** ^7^	53.4	28*** ^8^	50.0	1.148	0.550	2.395	0.713
Education level^2^	***		***					0.196
Elementary school or less	33	52.4	38	61.3				
Upper secondary school^2^	21*	33.3	19**	30.6	1.273	0.586	2.766	0.543
Post-secondary or university^2^	6***	9.5	5***	8.1	1.382	0.386	4.946	0.619
University degree^2^	3	4.8	0	0.0	NA	0.000	NA	0.990
Mother’s educational level^2^			**					0.062
Elementary school or less	11	17.5	12 ^4^	19.7				
Upper secondary school^2^	18	28.6	27 ^4^	44.3	0.727	0.264	2.002	0.538
Post-secondary or university^2^	8	12.7	10 ^4^	16.4	0.873	0.253	3.011	0.829
University degree^2^	26	41.3	12** ^4^	19.7	2.364	0.814	6.866	0.114
Father’s educational level^2^								0.436
Elementary school or less	22	34.9	16 ^4^	26.2				
Upper secondary school^2^	12*	19.0	19 ^4^	31.1	0.459	0.174	1.209	0.115
Post-secondary or university^2^	6*	9.5	6 ^4^	9.8	0.727	0.198	2.674	0.632
University degree^2^	23	36.5	20 ^4^	32.8	0.836	0.347	2.016	0.691
Occupation (studies or work; no/yes)	24/39***	38.1/61.9	19/43***	30.6/69.4	0.768	0.365	1.618	0.488
Steady relationship at death	18*	28.6	35	56.5	0.309	0.147	0.648	0.002
Addiction (no/yes)	32/31***	50.8/49.2	36/26***	58.1/41.9	1.341	0.662	2.717	0.415
Alcohol abuse	11**	17.5	8*	12.9	1.428	0.532	3.832	0.479
Substance abuse	20***	31.7	21***	33.9	0.908	0.430	1.917	0.800
Abuse of anabolic steroids	6	9.5	9	14.5	0.620	0.207	1.860	0.394
Abuse of psychoactive drugs	6*	9.5	20***	32.3	0.221	0.082	0.598	0.003
Alcohol or drugs at the time of death	37^4^	60.7	26 ^4^	42.6	2.075	1.008	4.273	0.048
Mother’s addiction (no/yes)	60/3	95.2/4.8	57/5	91.9/8.5	0.570	0.130	2.495	0.456
Father’s addiction (no/yes)	43/10**	68.3/31.7	46/16*	74.2/25.8	1.337	0.614	2.911	0.464
Investigated or sentenced for criminal acts	18	28.6	36***	58.1	0.267	0.126	0.566	0.001
Father’s criminality (investigated or sentenced)	3	4.8	3	4.8	0.983	0.191	5.070	0.984
Psychiatric care (no/yes)	18/45**	28.6/71.4	26/36	41.9/58.1	1.806	0.858	3.799	0.119
Outpatient child and adolescent psychiatry	28	44.4	30	48.4	0.853	0.422	1.725	0.659
Inpatient child and adolescent psychiatry	6*	9.5	8*	12.9	0.711	0.231	2.182	0.551
Outpatient adult psychiatric care	35***	55.6	17	27.4	3.309	1.567	6.985	0.002
Inpatient adult psychiatric care	25***	39.7	11**	17.7	3.050	1.338	6.955	0.008
Admission to treatment unit for young people	9**	14.3	12***	19.4	0.694	0.270	1.788	0.450
Mother’s psychiatric care (no/yes)	51/12	81.0/19.0	49/13	79.0/21.0	0.887	0.369	2.132	0.789
Mother’s outpatient psychiatric care	10	15.9	12	19.4	0.786	0.312	1.980	0.610
Mother’s inpatient psychiatric care	2	3.2	1	1.6	2.000	0.177	22.640	0.576
Father’s psychiatric care (no/yes)	48/15	76.2/23.8	54/8	87.1/12.9	2.109	0.822	5.411	0.120
Father’s outpatient psychiatric care	13	20.6	7	11.3	2.043	0.755	5.528	0.160
Father’s inpatient psychiatric care	4	6.3	2	3.2	2.034	0.359	11.530	0.423
Depression spectrum disorder	42***	66.7	22	35.5	3.636	1.738	7.608	0.001
Autism spectrum disorder (ASD)	11*	17.5	3	4.8	4.090	1.081	15.469	0.038
Autistic disorder (AD)	4	6.3	1	1.6	4.068	0.442	37.477	0.216
Attention deficit hyperactivity disorder (ADHD)	13	20.6	19	30.6	0.588	0.261	1.329	0.202
Conduct disorder (CD)	9	14.3	19*	30.6	0.377	0.155	0.917	0.031
Oppositional defiant disorder (ODD)	13	20.6	12	19.4	1.083	0.451	2.604	0.858
Borderline personality disorder (BPD)	25*** ^5^	43.9	21*** ^6^	40.4	1.153	0.538	2.471	0.714
Antisocial personality disorder (APD)	7 ^5^	12.3	19*** ^6^	36.5	0.243	0.092	0.643	0.004
Divorced or separated parents	38*	60.3	37	59.7	1.027	0.502	2.101	0.942
Dead parent^3^								0.062
Dead mother	3	4.8	1	1.6	4.049	0.805	20.369	0.090
Dead father	7	11.1	2	3.2	3.471	0.350	34.410	0.288
Dead both parents	2	3.2	0	0.0	NA	0.000	NA	0.988
Adverse childhood experiences (ACE)	M = 2.16***	SD = 1.55	M = 1.82***	SD = 1.52	1.156	0.916	1.458	0.222
Being bullied	28	44.4	15***	24.2	2.507	1.167	5.385	0.019
Being sexually assaulted	13**	20.6	1	1.6	15.340	1.938	121.395	0.010
Foster-home placement	4	6.3	6	9.7	0.633	0.170	2.361	0.496
Suicide attempt among relatives	20	31.7	18	29.0	1.111	0.517	2.386	0.787
Death by suicide among relatives	22	34.9	11	17.7	2.439	1.060	5.612	0.036
Recent death close to the young person (yes)	48	76.2	36	58.1	2.311	1.072	4.984	0.033
Stressful life events previous year (SLY; yes)	62	98.4	53	85.5	10.528	1.292	85.816	0.028
Severity of stressful life events (SRI)	M = 148.14***	SD = 56.33	M = 111.23***	SD = 58.87	1.011	1.005	1.018	0.001
Number of stressful life events (LEI)	M = 2.84***	SD = 1.05	M = 2.10***	SD = 1.07	1.965	1.352	2.856	0.000
Planful Problem-Solving	M = –0.56***	SD = 1.03	M = –0.08*	SD = 1.07	0.647	0.449	0.932	0.019
Seeking Social Support	M = 0.29*	SD = 1.01	M = –0.03	SD = 1.03	1.367	0.940	1.879	0.102
Escape-Avoidance	M = 0.55***	SD = 0.82	M = 0.24***	SD = 1.09	1.392	0.940	2.061	0.098
Confrontive Coping	M = 0.15*	SD = 1.07	M = 0.28**	SD = 1.14	0.893	0.638	1.250	0.510

*** *p* < .001

** *p* < .01

* *p* < .05 in comparison to the control group (yellow highlighted).

*Note*. ^1^ reference category “average results”; ^2^ reference category “elementary school or less”; ^3^ reference category “none”; ^4^ *N* = 61 owing to missing data; ^5^ *N* = 57 age >18 years; ^6^ *N* = 52 age >18 years; ^7^ *N* = 58; ^8^ *N* = 56; NA = not available due to too low frequency in at least one of the groups.

### Psychological autopsy interviews

In the suicide group, the interviews were conducted three to 13 months postmortem and in the SVD group three to 16 months postmortem. At least one interview per case was performed, preferably with the parents of the dead person, but siblings and occasionally other relatives could replace a non-participating parent, and in the control group also with the young person (105 interviews in the suicide group, 91 interviews in the SVD group, and 240 interviews in the control group). The semi-structured interview protocol followed basic procedures for psychological autopsy studies, investigating the background of suicide, the person’s state of mind, mental and physical health, personality characteristics, adverse life experiences, socioeconomic and educational background, and integration in the society.

### Variables of interest

#### Psychiatric diagnoses

The interviews comprised criteria for the following psychiatric diagnoses according to DSM-IV-TR [28]: Autistic Disorder (AD), Attention Deficit Hyperactivity Disorder (ADHD), Conduct Disorder (CD), Oppositional Defiant Disorder (ODD), depression spectrum disorder (Mood Disorder, Major Depressive Disorder, or Depressive Episode), Borderline Personality Disorder (BPD), and Antisocial Personality Disorder (APD).

#### Strains in life

Early strains in life were operationalized in terms of Adverse Childhood Experiences (ACE). This concept comes from the Centers for Disease Control and Prevention (CDC) Kaiser ACE Study, originally reported in 1998 [29]. The 10 ACEs measured in the present study were identical to those used in most recent ACEs studies [30], namely: abuse variables (emotional and verbal abuse, physical abuse, sexual abuse), neglect variables (emotional neglect, physical neglect), and household dysfunction variables (witnessing a mother being abused, household substance abuse, mental illness or depression in household, parental separation or divorce, imprisoned household member).

Recent strains in life were assessed in terms of stressful life events in the previous year, using all relevant interview information and scored following a modified non-adult version of the Holmes and Rahe Social Readjustment Rating Scale (SRRS) [31]. To the original 39 SRRS items we added these six age-relevant items: imprisonment, exposed to violence, moving away from home, increase in arguments with parents or partner, economic difficulties, and starting or interrupting work or studies. Each of the 45 items was ascribed a Life Change Unit (LCU) [32] on a 100-point scale. The *Social Readjustment Index* (SRI) is the sum of all LCU scores. We also calculated the *Life Event Index* (LEI) [33], which is simply the total number of stressful life events for each case.

#### Ways of coping

The interview protocol included the 24-item Shortened Ways of Coping Questionnaire (WCQ), one of the most frequently used coping scales [34]. In the present study, each yes-no response to the 24 WCQ items in each case was based on an aggregated yes-no response from all responders in each case. Factor analysis of the WCQ responses in 229 cases [23] gave a four-factor solution: Planful Problem-Solving, Escape-Avoidance, Seeking Social Support, and Confrontive Coping (aggressive, hostile acting-out), together explaining 54% of the variance in the material.

### Data analysis

In the preliminary step of the statistical analysis, univariate effects of demographic and potential risk factors on the dependent variables were tested separately for cases of suicide–control cases and cases of SVD–control cases, using logistic regression. Comparisons of the suicide and SVD groups with general controls were reported in our previous study [18], based on ANOVA and Tukey’s honestly significant difference (HSD) post-hoc test, and were now re-calculated applying the same statistical method for all potential risk factors, i.e., logistic regression. The present study adds a direct comparison between the suicide and SVD groups among variables significantly different from the control cases. A third logistic regression analysis was calculated to test univariate effects of demographic and risk factors on the dichotomous outcome of having died by suicide, rather than by SVD. Following this, we looked for factors similar for the cases of suicide and cases of SVD, but significantly different from the control cases. Lastly, logistic regression was also used to calculate coping by life events interaction effects on having died by suicide. Cause of death by coping (life events) interaction effects on life events (coping) were calculated with linear regression analysis.

## Results

Approximately half of those who died by suicide (50.8%; 54.5% females and 48.8% males) had made previous suicide attempts, whereas none of the females and only 10.9% of the males attempted suicide in the SVD group. Looking at distinguishing risk factors for the two target groups, identified in univariate analyses, we found 21 significant differences on variables significantly different from the control cases (yellow-highlighted in Table 1). Distinguishing for the suicide group was lower frequency of living in a steady relationship (28.6%), adult outpatient (55.6%) and inpatient (39.7%) psychiatric care, depression (66.7%), autism spectrum disorder (17.5%), being sexually assaulted (20.6%), higher frequency of recent stressful life events (98.4%), and lower levels of Planful Problem-Solving (M = -0.56). Distinguishing for the SVD group was a predominance of males (88.7%), younger mother (M = 27.1), lower elementary school results (30.6%), abuse of psychoactive drugs (32.3%), being investigated or sentenced for criminal acts (58.1%), conduct disorder (30.6%) or antisocial personality disorder (36.5%).

Looking at similarities between the suicide group and the SVD group, we found 16 risk factors common for both groups, but distinguishing both of them from the control group (yellow-highlighted in Table 1). Common risk factors included lower educational level, absence of work or studies, different forms of addiction, child and adolescent psychiatric care, borderline personality disorder, adverse childhood experiences, low levels of Planful Problem-Solving, and high levels of Escape-Avoidance and Confrontive Coping.

In regression analyses with group as a dichotomous predictor there was a significant difference between the suicide and the SVD group in Planful Problem-Solving (*b* = 0.416, *p* = 0.026) but not on the other three coping factors (*p* = 0.058, 0.168, and 0.523 for Escape-Avoidance, Seeking Social Support, and Confrontive Coping, respectively). Taken together, these analyses indicate that the suicide group had lower levels of the more adaptive coping strategy Planful Problem-Solving than the SVD group, but both groups had similarly high levels of the more maladaptive coping strategies (Escape-Avoidance and Confrontive Coping).

Differences in the association between coping and life events between the two groups were calculated through interaction effects (group × coping interaction effects on life events, and group × life events interaction effects on coping). We also calculated life events by coping interaction effects on group membership. None of these associations was significant, i.e., the association between coping and life events did not differ between the suicide and the SVD groups, and the association between group membership and coping (life events) did not differ depending on degree of life events (coping). Accordingly, pair-wise comparisons with the control group showed similar patterns for both target groups, distinguishing them from the control group. Differences in Planful Problem-Solving could be accounted to some degree by differences in ACEs, indicating similar mediated effects of ACE on the low levels of the more adaptive coping strategy Planful Problem-Solving in the suicide and the SVD group.

## Discussion

Our findings are congruent with several psychological theories of suicide [13]. The examination of coping strategies confirmed social problem-solving vulnerability both in the suicide group and in the SVD group, consistent with the theory of *cognitive rigidity in problem-solving* [35]. The examination of risk factors suggested that psychiatric illness per se might constitute a severe strain in life but lead to suicide mainly if combined with other vulnerability factors, as previously postulated by the *clinical-biological model of suicide* [36]. According to this model, a common trait factor associated with suicidality is responding to stressful life experiences with hostility and aggression [21]. The present study showed that this factor is common for cases of suicide and SVD but is more prominent in cases of SVD. Thus, suicide and SVD might be consequences of underlying aggressive impulses that, in combination with other risk factors, determine whether the aggression is directed toward others or toward oneself (*two-stage model of outward or inward directed aggression*) [37, 38].

**Fig 1:**
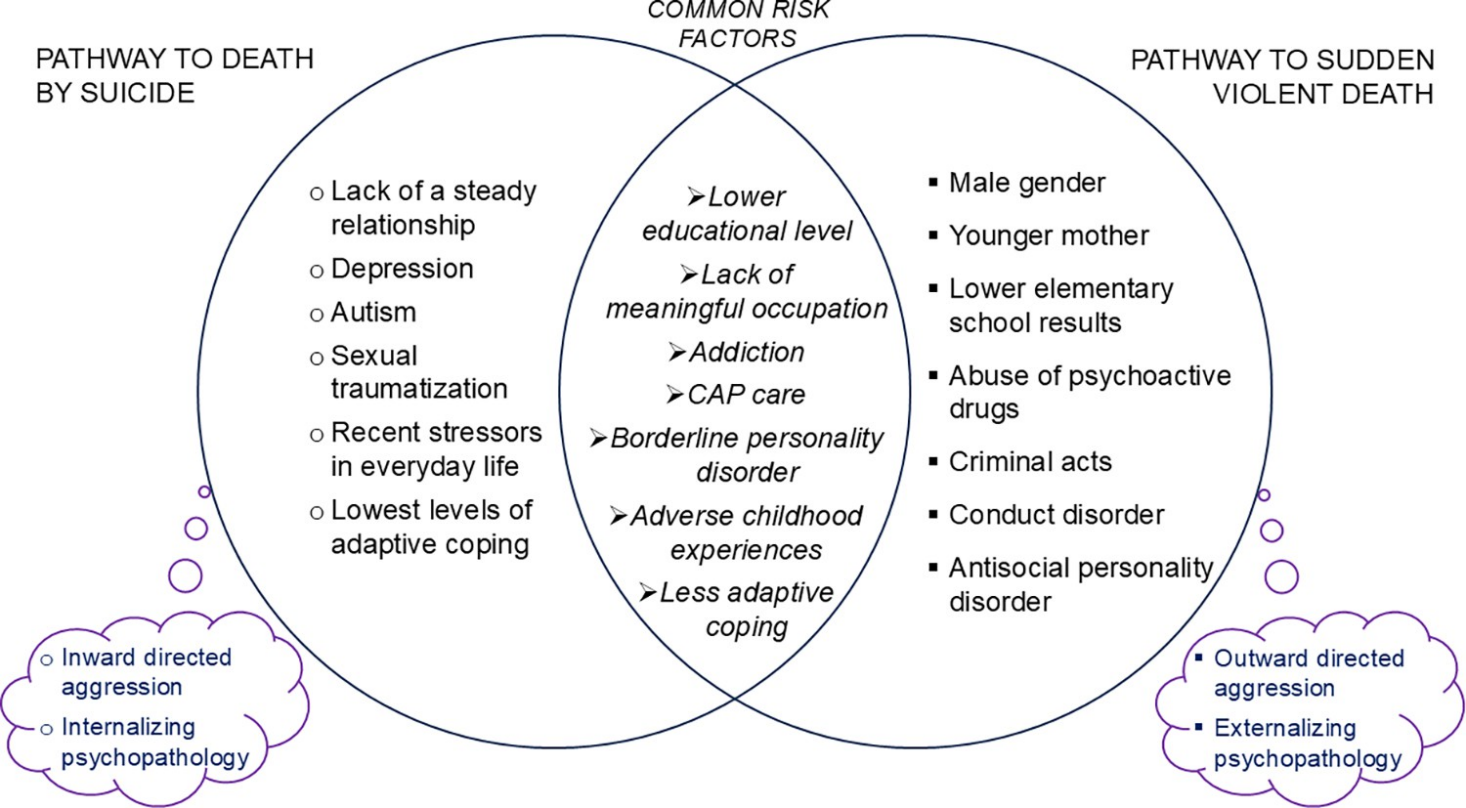
Common risk factors and distinct pathways to death by suicide and to SVD.

To summarize, the answer to our question “Two sides of the same coin?” must be: only partially. There are several common risk factors for both groups, distinguishing them from the control cases. But there are also several significant differences between the cases of suicide and SVD. In other words, there is a common ground of adverse childhood experiences, other vulnerabilities and strains in life, lower educational level and lack of work or studies, addiction, as well as less adaptive coping for both forms of premature violent death, but there is also a substantial divide for the two contrasting developments ending with suicide or with SVD, respectively. The pathway to death by suicide includes lack of a steady relationship, sexual traumatization, depression, autism, being psychiatric out- or inpatient, recent stressors in everyday life, lowest levels of adaptive coping, and use of alcohol and drugs in connection with suicide. The pathway to SVD includes male gender, younger mother, poor elementary education, acting out in form of criminal behavior, conduct disorder and antisocial personality disorder, together contributing to risk-taking behavior. The dividing line between the two pathways to premature unnatural death can be interpreted in terms of inward or outward directed aggression and internalizing versus externalizing psychopathology (Fig 1). However, it has to be noted that the path to sudden violent death seems to show some similarities to a third path: to destructivity and violence in interpersonal and intergroup relationships, criminal acts, and hurting others [39]. For example, young adult violent offenders might direct aggressive behaviors not only toward other people, but also toward themselves [40].

In the present study, approximately 29% of those who died by suicide and 42% of those who died a SVD, respectively, had never sought or never received any help from mental health services. This might suggest barriers to help-seeking among suicidal decedents, studied in a systematic review [41], and even higher barriers in the SVD group, probably due to the marked male predominance in this group, more of outward directed aggression and externalizing psychopathology (Fig 1). However, it should be noted that barriers against help-seeking are not only related to the young persons themselves but also include professionals, organization of school system, labor market, and routine mental health care.

On a theoretical level, the common feature of paths to suicide and to SVD is the existence of destructive and self-destructive processes. Based on our findings (Fig 1), common goals for prevention of both suicide and SVD should include focus on consequences of adverse childhood experiences; facilitating school learning and finding a job or alternative forms of meaningful occupation; promoting more adaptive coping strategies; addiction prevention and treatment; as well as routine follow-up of child and adolescent psychiatric contacts. Specific targets for suicide prevention should comprise focus on young people with lowest levels of adaptive coping, depression prevention and treatment, and paying attention to young people with autism. Specific targets for prevention of SVD should involve early recognition and response to learning difficulties, abuse of psychoactive drugs and delinquent behavior, paying attention to expressions of outward directed aggression, and effective treatment of conduct disorder and antisocial personality disorder. Ultimately, prevention of suicide and other forms of life-threatening behavior among young people may be facilitated by social, educational, and therapeutic interventions addressing feelings of powerlessness and loss of control over their lives, hostile contempt, violence in interpersonal and intergroup relationships, and externalization and projection onto others of own shortcomings and weaknesses.

### Strengths and limitations

The main assets of the present study include the use of multiple informants, and the focus on the partially common path and dividing developments for the two contrasting forms of premature unnatural death, as compared to living controls. Furthermore, the relatively low attrition (16–18%) may contribute to high representativity of our results. This should be compared with the usual dropout rate of 40–50% for psychological autopsy studies, reported in a review of methodological issues [42].

The most serious limitation of our study is the use of almost 20 years old archival data. Although we can hypothesize that some, if not most, of the risk factors associated with suicide and SVD, are still the same, including the use of less adaptive coping strategies, other factors might have been influenced in this period by, for example increased suicide awareness, greater focus on prevention, the use of social media and online interventions, experiences from the covid-19 close-down, climate crisis and the war in Europe, etc. This time gap may be a potential source of bias, and our results should be interpreted with caution as an initial exploration of potential similarities and differences between paths to suicide and to SVD. A further limitation is the wide age span (10–25 years), which includes different stages of the maturation process, and the limited number of cases below 18 years of age. Thus, there is a need of up-to-date replication studies in different sociocultural contexts and age groups.

Parents and other close relatives are usually considered as the most appropriate informants in cases of sudden death among young people, providing their attempts at in-depth understanding of what contributed to the lethal outcome. At the same time, they might not be aware of important information [42]. Another potential source of bias might be the search after meaning, trying to identify, in retrospect, circumstances that could explain the death [11]. The use of multiple informants minimized the risks. On the other hand, the procedure of weighing the informants’ answers inevitably involves a risk of subjective judgment. Another potential source of error is the varying number of informants in each case. A potential source of bias is the fact that the cases were deceased whereas the controls were not [27]. In the control group, living controls were included, in addition to their relatives, thus resulting in what Brent [43] called “asymmetry of informants” in psychological autopsy studies.

A further limitation is the large number of statistical comparisons, which increases the risk of type 1 error. However, a more rigorous significance criterion, such as a Bonferroni correction, would increase the risk for an overestimation of similarities between the two target groups, thus yielding incorrect answers to our research questions. Balancing the two risks, we decided to maintain the customary 5% significance level. Nevertheless, the results must be interpreted with caution.

### Implications

As usual, the present study raises new questions. Half of the cases of suicide had made no previous suicide attempt. What is common and what is different about these cases and those with single or multiple previous attempts? One tenth of those who died by SVD had also attempted suicide, which only is slightly more than in the control group. What is common to these cases and cases of death by suicide? Psychological autopsy studies can give us still deeper and highly relevant knowledge, for example of similarities and differences in processes ending in suicide or SVD. However, we also need more extensive and labor-intensive longitudinal research, focusing on interactions between different factors across time and on subgroup differences, including gender, different age groups, privileged and underprivileged housing environment, etc. Another important area of further research is changing the focus from risk factors and destructive processes to protective factors and adequate methods for creating benign circles. Such studies can contribute to more targeted prevention of both suicide and SVD. Furthermore, we urgently need studies of pathways to criminality and violent acts towards others.
